# Development of a functional cake with probiotics and micro-encapsulated essential oils: Evaluation of nutritional properties, liver protection, and immune boosting

**DOI:** 10.1016/j.heliyon.2023.e22918

**Published:** 2023-12-03

**Authors:** Manal M. Ramadan, Eman F. El Haggar, Rasha S. Mohamed, Khaled F. Mahmoud, Ahmed M. Mabrouk, Amal G. Hussien, Abeer E. Mahmoud, Ola A.M. Mohawed, Tamer M. El-Messery

**Affiliations:** a Chemistry of Flavors and Aromas Department, National Research Centre, Dokki, Cairo, Egypt; b Nutrition and Food Science Department, Faculty of Home Economics, Arish University, Egypt; c Nutrition and Food Science Department, National Research Centre, Dokki, Cairo, Egypt; d Food Technology Department, National Research Centre, Dokki, Cairo, Egypt; e Dairy Department, National Research Centre, Dokki, Cairo, Egypt; f Biochemistry Department, National Research Centre, Dokki, Cairo, Egypt; g Hormones Department, National Research Centre, Dokki, Cairo, Egypt; h International Research Centre “Biotechnologies of the Third Millennium”, Faculty of Biotechnologies (BioTech), ITMO University, St. Petersburg, 191002, Russia

**Keywords:** Innovative cake, Probiotics, Sweet potatoes, Clove oil, Cinnamon oil, Thioacetamide, Immunosuppression and liver protection

## Abstract

This study used probiotics and micro-encapsulated clove and cinnamon oils to develop a functional cream-stuffed cake based on sweet potatoes flour and rice flour instead of wheat flour. The cake was evaluated for its physical, chemical, and sensory properties and its antioxidant capacity. The protective effect of the cake against liver injury and immunosuppression induced by thioacetamide injection in male rats was also evaluated. The study found that eugenol and cinnamaldehyde were the majority of volatile compounds in the essential oils used in the cake, with values of 78.73 % and 81.57 %, respectively, as determined by GC-MS analysis. The viable counts of added probiotics in the cake ranged from 13.15 to 11.21 log CFU/g and were still above the threshold for health benefits. The cake had an increased dietary fiber and protein content while containing a low-fat percentage compared to a commercial cake sample. The innovative cake also contained higher levels of water-soluble and fat-soluble vitamins and minerals such as iron, calcium, potassium, and zinc. The antioxidant capacity of the cake was evaluated, and it was found to contain 1827.23 mg GAE/100 g of total phenols and 97.13 mg QE/100 g of flavonoids. The cake was also found to have antioxidant activity and was effective in protecting the liver from oxidative stress and inflammation and reducing immunodeficiency associated with liver damage.

## 1 Introduction

Developing a functional cake with probiotics and micro-encapsulated essential oils is a novel and urgent area of research due to the potential health benefits such a product could provide. Probiotics are live microorganisms that confer health benefits when consumed in adequate amounts and have been associated with improved immune function, gut health, and other health outcomes [1,2]. Essential oils are volatile compounds derived from plants that have been shown to possess antimicrobial activity against both gram-positive and gram-negative bacteria [3] and antioxidant and anti-inflammatory properties [4]. The micro-encapsulation of essential oils could enhance their stability and bioavailability and protect them from oxidation and degradation during processing and storage [5]. Population growth around the world causes a longer life expectancy due to a decrease in natural resources, especially wheat flour, and due to the economic crisis that the world is going through from wars and the spread of the coronavirus, in addition to the health problems experienced by some groups that are sensitive to various kinds of food [6]**.** Wheat flour contains gluten, and to meet the increasing consumer demands, sustainable and efficient production processes are being developed to reduce or limit the use of wheat flour. The search for low-cost, economical, healthier, and safer alternatives that have functional properties in protecting the liver [7]

Maize, cassava, sweet potato, and yams are significant sources of food starch, while maize, barley, rice, etc., act as a secondary source of starch in different parts of the world. The original or raw starch occurs in the form of granules and thus affects the physical and chemical properties of the final product. The choice of rice and sweet potatoes was one of the best gluten-free sources, which adds stability to the product, the perfect size, and the absence of cracks on the surface of the cake. The granules' size, shape and molecular arrangement also depend on the plant species and genetic environment interactions. The starch biosynthetic pathway generally leads to forming two types of glucose polymers, linear amylose and highly branched amylopectin. In addition, other minor components of starch, such as proteins and fats, may be present [8].

Nanoparticle systems have the potential to provide numerous health benefits, particularly in medicine. Nanoparticles are tiny particles with dimensions on the nanometer scale (typically less than 100 nm). Due to their small size, they can penetrate tissues and cell membranes more quickly than larger particles, which makes them ideal for drug delivery applications [9,10]. One of the main advantages of nanoparticle systems for drug delivery is that they can selectively target specific cells or tissues in the body [[11], [12], [13]]. It can improve the efficacy of drugs and reduce side effects by minimizing exposure to healthy tissues. For example, cancer drugs can be encapsulated in nanoparticles that target only cancer cells, improving treatment effectiveness while reducing toxicity to healthy tissues [14,15].

The liver is an important organ in both the human and animal body. It oversees several processes, including those that assist immunity, digestion, detoxification, and vitamin storage; around 2 % of an adult's body weight comprises it. The liver is a prominent organ due to its involvement in the blood supply to the body from the portal vein (about 75 %) and the hepatic artery (about 25 %) [16]. The liver can be destroyed for various causes: fat intake, exposure to pollutants, heavy metals, mycotoxins, and other chemicals. The liver has numerous distinct immunological characteristics, including the ability to induce inherent immune immunity, tolerance, poor hematopoiesis and the adaptive immune response against overreactive autoimmune in the fetus's liver. Despite the liver's principal tasks not typically thought of as immune-related, the liver also performs various vital immunological functions. For instance, hepatocytes produce 80–90 % of the circulating innate immunity proteins in the body, and the liver contains many resident immune cells [17]. Accordingly, any damage to the liver may negatively affect the body's immune status.

Probiotics are living microbes that boost the host's health when given in sufficient quantities [18]. In rats exposed to liver illness, probiotic administration reduces liver oxidative stress, inflammation, and fibrosis and is considered a safe and effective alternative treatment [19]. Gut microbes significantly influence the health of the liver. As a result, altering gut microbiota offers a viable strategy for hepatoprotection. Probiotics can treat liver damage by reestablishing a healthy gut microbiome. Probiotics can both boost host immunity and induce the synthesis of antimicrobial peptides [20,21].

Clove and cinnamon essential oils, with unique aromatic compounds, might be promising and practical for immune system infections and have good oral bioavailability. In addition, they are considered safe dietary supplements for immune-compromised patients [22]. The immune system may benefit from cinnamon essential oil as a flavouring addition to help it fight viral illnesses like SARS-CoV-2 [23]. According to a recent study, clove oil interacts with the SARS-CoV-2 spike protein and significantly reduces the entry of pseudo-type SARS-CoV-2 into human HEK293 cells that carry the ACE2 gene.

Moreover, the mechanism behind eugenol's - the primary ingredient in clove oil - ability to alter the levels of NF-kB, IL-6, IL-1, and TNF in human lung cells involves its ability to inhibit the activation of NF-kB. This transcription factor regulates the expression of genes involved in the inflammatory response. Eugenol inhibits the phosphorylation and degradation of IκBα, an inhibitor of NF-kB, leading to the accumulation of IκBα and the suppression of NF-kB activation. It, in turn, leads to the downregulation of pro-inflammatory cytokines like IL-6, IL-1, and TNF. Therefore, eugenol's anti-inflammatory effect was mediated by its ability to inhibit NF-kB activation [24].

Therefore, the study aimed to produce an innovative healthy cake with functional properties consisting of rice flour and sweet potato flour flavoured with micro-capsules of clove and cinnamon oils and probiotic cream to study the nutritional values of a gluten-free product, its physical and chemical properties, and antioxidants activity to examine the hepatoprotective effect of the innovative cake against liver injury and the ensuing immunosuppression induced by repeated thioacetamide injections in male rats.

## 2 Materials and methods

### 2.1 Materials

#### 2.1.1 Raw materials

The raw materials used in preparing the cake (rice flour, sweet potatoes flour, eggs, vanilla, baking powder, cinnamon, and clove powder), in addition to the commercial cake, were obtained from a local market in Egypt. Casein which was obtained from Scerma Co., France. Gum Arabic (GA), maltodextrin (MD) and Tween®80 were purchased from Aromsa Co., Kocaeli, Turkey.

#### 2.1.2 Chemicals

DPPH (2,2-diphenyl-1-picrylhydrazyl), the ferric reducing/antioxidant power (FRAP), ferric chloride, potassium ferricyanide and sodium phosphate buffer (pH 6.6) were obtained from Sigma-Aldrich (St. Louis, MO). All chemicals were of analytical grade.

#### 2.1.3 Probiotic strains

The probiotic strain Lactobacillus acidophilus CH-2 was obtained from Chr. Hansen's Lab., Denmark, and Lactobacillus plantarum NRC AM10 were isolated, characterized, and identified by Jantararussamee et al. [19].

### 2.2 Methods

#### 2.2.1 Probiotic suspension preparation

Probiotic strains were cultivated individually in conical flasks containing 1000 mL MRS broth and incubated at 37 °C for 48 h; the cells were then collected by centrifugation at 5000 rpm for 15 min at 4 °C to obtain probiotic biomass. The cell biomass was washed twice with sterilized physiological saline and centrifuged in the same conditions to remove the residual medium. The pure biomass was resuspended in sterilized distilled water (1:1 w/v) containing 10^9^–10^10^ CFU/ml and kept at 5 °C for manufacturing functional cake.

#### 2.2.2 Isolation of clove and cinnamon essential oil

Using the Clevenger apparatus and hydro-distillation process, clove and cinnamon oils were extracted for 3 h [25]. The yield of the volatile oils was measured in grams and 100 g^−1^ dry plants.

#### 2.2.3 Gas chromatography-mass spectrometry (GC–MS) analysis of clove and cinnamon essential oil

Analysis was performed using gas chromatography coupled to a mass spectrometer (Hewlett-Packard model 5890 and 5970, respectively). Volatiles were separated using a fused silica capillary column DB-5 (60 m × 0.32 mm i.d. × 0.25 μm film thickness). The oven temperature was kept at 50 °C for 5 min, then raised by 4 °C/min from 50 to 250 °C. As the carrier gas, helium was employed at a 1.1 ml/min flow rate. The pure oil (20 μL) was dissolved using 1 ml diethyl ether. The split ratio was 1:50, the sample volume was 1 μL, and the injector temperature was 220 °C. At 70 eV, electron impact mode (EI) mass spectra were obtained with scan *m*/*z* ranging from 39 to 400 amu. The isolated peaks were identified by comparing the isolated peaks to those of authentic compounds and published data, as reported by Adams [26], as well as to mass spectra library data from the National Institute of Standards and Technology (NIST).

#### 2.2.4 Preparation of nanoemulsions with clove and cinnamon oils

Gum Arabic (GA) 15.0 % w/v was hydrated in deionized water, then used to create the emulsion. To allow for complete hydration, leave the gum solution at 4 °C overnight, then add 15 g of maltodextrin (MD) to the gum solution to dissolve. Tween®80 (1.0 % w/v, based on water) was added. To obtain oils/total solids emulsion with a ratio of 1:3, vigorous mixing was done first, and then a specific weight of oils was added. 10.0 g of oils, 15.0 g of GA, 15.0 g of MD, 1.0 g of Tween 80, and 100 mL of water make up the emulsion. The mixtures were homogenized using an Ultra-Turrax homogenizer (T18basic IKA, Wilmington, USA) at 18.000 rpm for 5 min. Finally, nanoemulsions were obtained by probe sonication, using an instrument from Sonics and Materials, Inc. Vibra-Cell with a power of 160W, a frequency of 20 kHz, and a 50 % pulse. The emulsion vessel was submerged in a cold-water bath to prevent the emulsion temperature from rising during ultrasound homogenization above ambient.

#### 2.2.5 Characterization of nanoemulsions

##### 2.2.5.1 Particle size, polydispersity index (PDI) and ζ-potential

The emulsion was characterized by evaluating its particle size, polydispersity index (PDI) and ζ-potential distribution. The ζ-potential was determined using a particle charge titration analyzer called Stabino® (Microtrac Europe, Montgomeryville, PA, USA). The particle size distribution was analyzed using the Mastersizer MS2000 instrument (Malvern Instruments, Worcestershire, UK).

##### 2.2.5.2 Transmission electron microscopy (TEM)

The morphology of the nanoemulsions was examined and visualized using transmission electron microscopy (TEM) with a JEM-2100 Electron Microscope (Instruments, China). Prior to imaging, the samples were coated with a thin layer of gold using the DST3 Nanostructured Coating Co. (Tehran, Iran) to facilitate electron microscopy.

##### 2.2.5.3 Atomic force microscopy (AFM)

The Atomic force microscopy (AFM) studies of nanoemulsions, samples were prepared by placing a droplet of the nanoemulsions suspension on a freshly cleaned mica sheet, allowing it to air-dry [11]. The observation and imaging of the samples were carried out using an AFM instrument (AFM coupled with WiTec alpha 300R Raman Imagining Microscope. The AFM images were measured in AC mode) in both amplitude and tapping modes.

#### 2.2.6 Dehydration of nanoemulsions with spray-drying

A spray dryer was used throughout the procedure to dry the prepared emulsion. An oil-in-water emulsion was created using spray drying as a first step in microencapsulation. According to Balci‐Torun [27], The two-fluid nozzle used in the spraying system had an external ring with a 1.5 mm opening and an internal tip with a 0.5 mm opening (Büchi Mini Spray Dryer B-290, Switzerland). Additionally, a drying air flow rate of 80 % of the suction fan controller was one of the constant process factors. The temperatures at the inlet and outlet were 160 ± 5 °C and 80 ± 5 °C, respectively. In addition, the powder produced was removed from the cyclone and the wall of the drying chamber.

#### 2.2.7 Characterization of encapsulated powder

##### 2.2.7.1 The encapsulation efficiency (EE)

The total oil content (TOC) of the microcapsules was determined by distilling 5 g of powder that was dissolved in 150 mL of distilled water for 3 h using a Clevenger-type apparatus. To extract the essential oil from the water phase, 2 mL of ethyl ether (VETEC, São Paulo, Brazil) was added. The solution was gradually heated to a boiling point and allowed to distill. The collected oil was gathered in a pre-weighed Erlenmeyer flask, and the ethyl ether was left to evaporate at room temperature for 24 h [28].

To determine the surface oil content, a modified method described by **El-Messery et al.** [29] was employed. In this method, 2 g of powder was mixed with 20 ml of hexane and stirred at 300 rpm for 10 min. The resulting suspension was filtered through a dried cellulose filter and washed three times with 20 mL of hexane. The filter was then kept in a vacuum desiccator until a constant weight was achieved by vaporizing any residual solvent. Finally, the oil encapsulation efficiency (EE) was calculated using Equation (2).

(1) $$\text{EE}\;\left(\%\right)=\frac{\;Total\;oil\;\text{content}\;-\;Surface\;oil}{Total\;oil\;\text{content}\;}\;X100$$

##### 2.2.7.2 Surface morphology of encapsulated powder

Scanning Electron Microscopy (SEM) using a Quanta FEG 250 SEM instrument (ThermoFisher Scientific, Oregon, USA) was employed to examine the morphology of dried nanoemulsions. To facilitate SEM imaging, the clove and cinnamon oil samples were coated with a layer of gold by mounting them on aluminum stubs using double-sided adhesive tape, and an Edwards sputter coater S150A (Crawley, England) was used for the gold coating. SEM was utilized to visualize the powdered forms of clove and cinnamon oils, providing insights into their morphology. The SEM analysis of the powder samples involved using an accelerating voltage of 20 kV, a working distance of 12.1 mm, and magnifications of 3000x and 6000x for clove and cinnamon oils encapsulated powder, respectively.

#### 2.2.8 Flavoured innovative cake preparation

According to the method of cake preparing that described by Bennion and Bamford [30], Innovative cake was prepared with the following ingredients: orange sweet potato (500 g), black honey (50 g), salt (1 g), fresh whole egg about (250 g), rice flour (250 g), baking powder (10 g), vinegar apple (0.05 ml), micro-encapsulated cinnamon oil (5 g), and micro-encapsulated clove oil (5 g). The cake formula was mixed and poured into baking pans that were baked, in a preheated oven, at 160 °C for 25–30 min, then cooled on racks for about 1 h. The cake was stuffed with full cream (200 g) fortified with probiotics suspension (10 g/100 g full cream). The stuffed cakes were packed in polypropylene bags and stored at fridge temperature for physicochemical and sensory evaluation analyses.

#### 2.2.9 Preparation of cake extract

To prepare the extracts, 20 g from cake samples was added to 200 mL ethanol (70 %, v/v), then thawed and stirred well at 200 rpm for 5 min. The solution was homogenized using an ultrasonic water bath for 5 min. Then, the mixture was filtrated using micro centrifugation for 10 min at 13,000 rpm. The supernatants were used for further analyses.

#### 2.2.10 Proximate composition of innovative and commercial cake samples

##### 2.2.10.1 Nutrition Facts of innovative and commercial cake samples

Using the Soxhlet extraction method, the total fat content and moisture were determined; the Kjeldahl method was used to determine crude protein (N × 6.25). The total ash of samples was determined according to Horwitz [31]. Carbohydrate content was estimated by subtracting the sum of crude fat, moisture, ash contents, and crude protein. Total sugar and calorie content were determined according to Rangnna [32] and Atwater [33], respectively.

##### 2.2.10.2 Total fat content

The total fat content of samples was carried out using the Soxhlet extraction method, according to **Horwitz** [31]. Petroleum ether was used to extract 5 g of dried sample throughout 6 h in a Soxhlet extraction device. The Petroleum ether was entirely vaporized from the containers after the extract of ether was filtered into them, and the rise in the volume of the container characterized the amount of fat present; the fat content (%) was calculated using equation (1):

(2) $$\text{Percent}\;\text{of}\;\text{fat}\;\text{content}\;\left(\%\right)=\frac{W2-W1}{W}\times 100$$

Where: W is sample weigh (g); W1 is empty container weight (g) and W2 is empty container weight + fat content (ether extract).

##### 2.2.10.3 Determination of dietary fiber

Total dietary fibre was analyzed by enzymatic methods [34]**.** The enzymes used in the analysis were α-amylase, protease, and amyloglucosidase, which were supplied at specific concentrations. The dietary fibre was calculated using Equation (2):

(3) $$\text{Dietary}\;\text{Fiber}\;\left(\%\right)=\frac{\;\left[\left(R1+R2\right)/2\right]\;–\;P\;–\;A\;-\;B}{\left[m1+m2\right]/2}$$

where: R1 = residue weight 1 from m1; R2 = residue weight 2 from m2; m1 = sample weight 1; m2 = sample weight 2; A = ash weight from R1; p = protein weight from R2 and B = blank.

##### 2.2.10.4 Minerals and vitamins content in cake samples

Using performing HPLC analysis an Agilent 1260 series [35], the vitamins A and C were determined in cake samples; Kromasil 100-5-C18 (4.6 mm × 250 mm ID, 5 μm) was performed to separate the components. The flame photometry method determined sodium and potassium as minerals in samples. Flame atomic absorption spectrometry determined magnesium, calcium, phosphorous, manganese, iron, and zinc [36].

#### 2.2.11 Total phenolic content (TPC) and total flavonoid content (TFC)

**Mythili et al.,** [37] determined the TPC of cake samples using the Folin-Ciocalteau reagent. The cake samples were first dissolved in methanol and then extracted. The extracted sample was mixed with Folin-Ciocalteau reagent and sodium carbonate, and the resulting mixture was left to incubate for 15 min. The TPC was then determined using an automated UV-VIS spectrophotometer at 765 nm and calculated using a Gallic acid calibration curve. The results are expressed as equivalents to Gallic acid, in milligrams of Gallic acid equivalents per gram of dry extract.

The total flavonoid content (TFC) of cake samples was estimated using a method described by **Mythili et al.** [37]**.** The cake samples were first dissolved in methanol and then extracted. The extracted sample was mixed with methanol, aluminium chloride, potassium acetate, and distilled water. The mixture was left at room temperature for 10 min, and the absorbance was measured at 415 nm using a UV/visible spectrophotometer. The TFC was then determined using a quercetin calibration curve, expressed in milligrams of quercetin equivalents per 100 mL.

#### 2.2.12 Antioxidant activity of cake samples

The antioxidant activity of cake samples was evaluated using two methods. The first method was the DPPH radical scavenging assay which measures the ability of the cake samples to scavenge free radicals. The second method was the Ferric reducing/antioxidant power (FRAP) assay which measures the reducing power of the cake samples. Both methods were performed according to the method described by **Sharopov et al.** [38]**.**

The DPPH radical scavenging assay was performed by dissolving 0.2 g of each sample in 25 mL of methanol and filtering the extract. One millilitre of the extract was mixed with the DPPH solution, and the mixture was left to incubate for 30 min. The absorbance of the solution was then measured spectrophotometrically at 517 nm. The percentage of DPPH radical scavenging activity was calculated using Equation (3),

(4) %DPPHradicalscavengingactivity=(Ac–As)/Ac×100

where As is the absorbance of the sample and Ac is the absorbance of the control in the absence of the sample.

The Ferric reducing/antioxidant power (FRAP) assay was performed by combining 2.5 mL of the cake extract solution with sodium phosphate buffer, potassium ferricyanide, and trichloroacetic acid. The mixture was centrifuged, and the upper layer was mixed with deionized water and ferric chloride. The absorbance values of the solutions were then read spectrophotometrically at 700 nm.

#### 2.2.13 Sensory evaluation of cake samples

Sensory evaluation of the cake samples was performed by a panel of 20 specialists in baking technology at a testing area. The sensory profile of the sponge cake was evaluated based on sensory attributes, as described by **Lawless and Heyman** [39]**.**

#### 2.2.14 Bacteriological analysis

Cake samples (10 g) were homogenized in 90 ml of physiological sterile saline (0.85 % NaCl w/v) in a water bath (40 °C) then the appropriate serial dilutions were prepared [40]. The viable probiotic counts in the cake were enumerated using the pour plate technique and MRS agar (Oxoid), according to **Hamdy et al.** [41]**.** The plates were incubated anaerobically at 37 °C for 48h. The microbiological results were expressed as log colony-forming units per gram (CFU/g).

#### 2.2.15 Biological evaluations

##### 2.2.15.1 The protective effect of the innovative cake against liver injury induced by thioacetamide

The experiment was done on male rats (Sprague Dawley white albino). The rats were housed individually in cages under hygienic conditions, in a temperature-controlled room of 25^○^C, and fed on a stock diet for one week for adaptation. The Standard control diet was formulated according to **Reeves et al.** [42], and it was nutritionally adequate (AIN-93A).

Thirty rats were included in the experiment, and their body weight ranged between 120 ± 10 gm. The animals in the experimental groups received twice weekly for eight weeks (200 mg/kg b.w., i.p.) of TAA [43]. These rats were classified into three groups (each of ten rats) as follows:

G1; rats were fed a standard diet, set as control negative.

G2; rats were fed a standard diet with TAA injection and set as control positive.

G3; rats were fed a standard diet supplemented with 35 % of the prepared Innovative cakes with TAA injection.

The food and water were allowed ad libitum. The consumed food was calculated as the difference between the weights of food admitted and that remaining or scattered within 24 h. The body weight of rats was followed twice a week. The experiment lasted for eight weeks. At the end of the experiment, the rats were fasted overnight. On the second morning, each rat was weighed, slightly anaesthetized, and blood samples were withdrawn over heparin and without heparin. The samples were centrifuged at 3500 rpm for 15 min to separate plasma and serum, which were stored at −40 °C till further analysis. The Ethical Committee for Medical Research of the National Research Centre in Egypt approved the experimental protocol (Code No. 19182.)

##### 2.2.15.2 Biochemical analysis

A portion of the whole blood was analyzed for haemoglobin levels using the **Drabkin** [44] method, while the remaining blood was centrifuged to obtain serum that further analysis for fasting blood glucose levels, the activities of alkaline phosphatase, aspartate transaminase, and alanine transaminase according to **Trinder** [45]**, Rheinhold & Seligron** [46] **and Reitman** [47], respectively. The levels of albumin, creatinine, and urea were determined using methods described by **Doumas et al.,** [48]**, Larsen** [49] **and Fawcett &Scott** [50]**,** respectively. The liver was homogenized and analyzed for MDA, GPx, CAT, and SOD activity using methods described by **Ohkawa et al.** [51]**, Paglia &Valentine** [52]**, Beers & Sizer** [53] **and Nishikimi et al.** [54]**,** respectively. Plasma T-Ch, HDL-Ch, LDL-Ch, and TG were determined using methods described by **Watson** [55]**, Burstein et al.** [56]**, Schriewer et al.** [57] **and Megraw et al.** [58]**,** respectively. Serum CD4, CD8, INF-ᵧ, and IL-6 were analyzed using enzyme-linked immunosorbent assay kits.

#### 2.2.16 Statistical analysis

Statistical analysis was accomplished via the one-way analysis of variance ANOVA followed by Duncan's test using SPSS version 16. The results were expressed as mean ± standard error (SE). The statistical significance of the difference was taken as P ≤ 0.05.

## 3 Results and discussion

### 3.1 Volatile compounds of clove and cinnamon essential oils

Table 1 illustrates the identified volatile compounds of clove essential oil using GC-MS. The major components were eugenol (78.73 %), eugenol acetate (11.52 %) and β-caryophyllene (6.03 %). **Haro-González et al.,** [59] also found that eugenol is the primary compound, accounting for at least 50 %. The remaining 10–40 % comprises eugenyl acetate, β-caryophyllene, and α-humulene. Clove oil is a fragrant plant extensively grown in tropical and subtropical regions and contains various volatile compounds and antioxidants, such as eugenol, β-caryophyllene, and α-humulene. Its essential oil is in high demand and widely used in various industries, including perfumery, cosmetics, healthcare, medicine, food, and flavouring. The Clove essential oil has been found to possess numerous biological activities that can have a positive impact on human health, such as exhibiting antimicrobial, antioxidant, and insecticidal properties [59].

**Table 1:** Volatile compounds of clove essential oil using GC-MS.

Peak No.	Components	Area %	Retention time (RT) (min)
1	Eucalyptol	0.3 ± 0.01	9.81
2	Terpinen-4-ol	0.23 ± 0.01	14.74
3	Methyl salicylate	0.82 ± 0.01	15.33
4	Chavicol	0.31 ± 0.01	17.29
5	Eugenol	78.73 ± 2.05	21.18
6	Copaene	0.31 ± 0.01	21.48
7	E)-Methyl cinnamate	0.32 ± 0.01	21.67
8	β-Caryophyllene	6.03 ± 0.15	22.87
9	α-Humulene	0.88 ± 0.02	23.89
10	Eugenol acetate	11.52 ± 0.11	26.15
11	Caryophyllene oxide	0.54 ± 0.01	27.77

The data are stated as average ± standard error.

Table 2 illustrates the identified volatile compounds of cinnamon essential oil using GC-MS. Cinnamaldehyde (81.57 %) and acetyl mercaptan (8.73 %) were major components. **Xavier et al.** [60] reported that cinnamon essential oil has biological activities, including anticancer, antidiabetic, antioxidant, anti-inflammatory and anti-human immunodeficiency virus. Such effects may be attributed to the unique secondary metabolic, including cinnamaldehyde, eugenol, cinnamyl acetate, methyl cinnamate, (E)-caryophyllene, and linalool. In a recent study by **Mutlu et al.** [61]**,** the aromatic constituents of cinnamon oil were analyzed using GC-MS. The results revealed the presence of 17 volatile components in the cinnamon leaf oil samples. The most abundant compounds identified were E-cinnamaldehyde (72.98 %), benzyl benzoate (4.01 %), β-caryophyllene (3.45 %), and *trans*-cinnamyl acetate (3.36 %) in the cinnamon leaf oil.

**Table 2:** Volatile compounds of cinnamon essential oil using GC-MS.

Peak No.	Components	Area %	Retention time (RT) (min)
1	Acetyl mercaptan	8.73 ± 0.13	3.642
2	Cinnamaldehyde	81.57 ± 3.51	17.959
3	Eugenol	1.36 ± 0.05	20.774
4	trans-Methyl cinnamate	1.43 ± 0.04	21.592
5	Ethyl Vanillin	1.51 ± 0.09	23.864
6	Vinyl *trans*-cinnamate	3.17 ± 0.16	32.247
7	iso-Butyl cinnamate	2.23 ± 0.09	32.481

The data are stated as average ± standard error.

### 3.2 Characterization of the nanoemulsions clove oil (NeClO) and cinnamon oil (NeCiO)

#### 3.2.1 Polydispersity index (PDI), particle size and ζ-potential

The Malvern Zetasizer Nano Z instrument was utilized to determine the polydispersity index (PDI) and mean droplet sizes of the nanoemulsions containing different oils. The PDI values indicate the degree of homogeneity in the distribution of oil droplet sizes, with values closer to zero indicating a more uniform distribution [62]. The PDI values for NeClO and NeCiO were reported as 0.080 ± 0.09 and 0.196 ± 0.09, respectively.

**Fig. 1:**
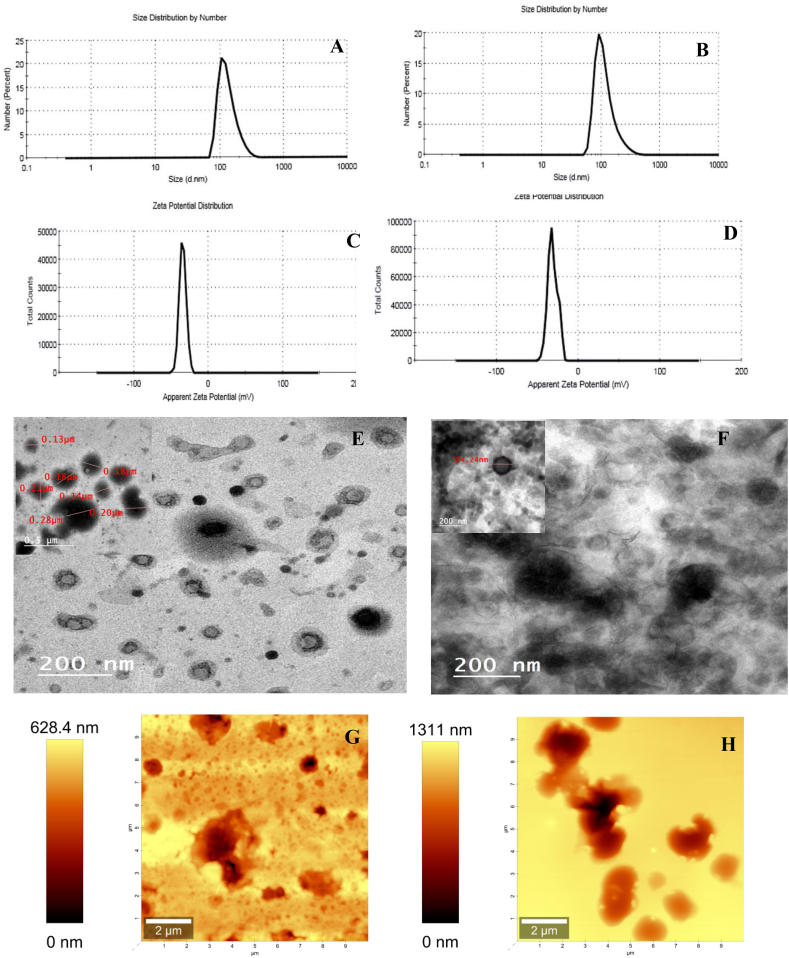

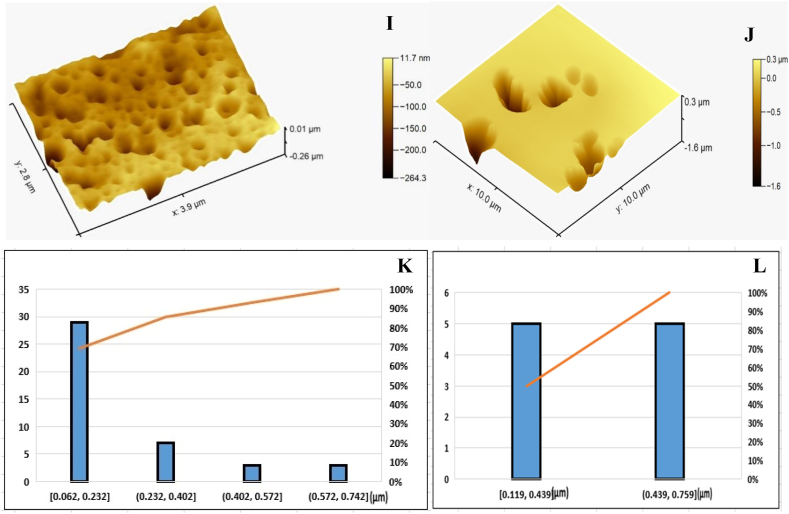
Characterization of nanoemulsions: Particle szie of NeClO **(A) and** NeCiO **(B).** ζ-potential of NeClO **(C) and** NeCiO **(D)**. Transmission electron microscopic (TEM) images of NeClO **(E) and** NeCiO **(F)**. Atomic force microscopy (AFM), 2D images of NeClO **(G) and** NeCiO **(H)**, 3D images of NeClO **(I) and** NeCiO **(J)** and Histogram representations of mean diameter of NeClO **(K) and** NeCiO **(L).**

By subjecting the emulsions to high-pressure homogenization at 18,000 rpm for 5 min, nano-sized droplets were formed using probe sonication (160 W power, 20 kHz frequency, 50 % pulse), and the diameter of the NeClO and NeCiO droplets were 132.20 ± 2.26 nm and 118.60 ± 3.61 nm, as shown in Fig. 1A, B respectively. It is important to note that the size of droplets in the nanoemulsions produced through probe sonication can vary depending on factors such as the interaction chamber, operating conditions, and emulsion composition [63]**.**

The ζ-potential is a measurement that indicates the degree of charge attraction or repulsion between particles. In the case of emulsions, having a significant ζ-potential helps prevent droplet coalescence and contributes to the stability of the emulsion [63]. The ζ-potential measurements were recorded for nanoemulsion droplets containing different oils. The ζ-potential values for the NeClO was found to be −31.30 ± 1.34 mV, while the NeCiO had a ζ-potential of −34.20 ± 1.66 mV. as shown in Fig. 1C, D respecticely. These values indicate a negative charge on the outer surface of the droplets, which is attributed to the presence of carboxylate groups introduced by the use of GA [64]. GA consistently exhibits a negative ζ-potential regardless of the pH due to the carboxylate groups it contains.

Emulsions with ζ-potential higher than +30 mV or lower than −30 mV are generally considered to be electrostatically stable [63]. Previous studies have reported that nanoemulsions containing 2%–8% oil exhibit negative zeta potentials ranging from −25.0 to −48.8 mV, and these emulsions showed good stability without significant effects on the oil [65]. The negative ζ-potential observed in this study can be attributed to the dispersion of nanoemulsion droplets, which contributes to the overall emulsion stability [66]. These ζ-potential results align well with the observed emulsion stability and are consistent with previous findings on the behavior of MD/GA [67]. In this ζ-potential range, MD and GA form a desired complex on the emulsion surface, leading to repulsion between droplets and preventing clotting and creaming of the encapsulated droplets [67].

#### 3.2.2 Transmission electron microscopy (TEM)

The TEM analysis of single-layer emulsions allowed for the examination of their microstructure, as shown in Fig. 1. In Fig. 1E and F, the morphology of NeClO and NeCiO droplets, respectively, coated with GA in the primary emulsions demonstrated a distinct spherical shape, with a clearly defined interface corresponding to the GA layer. The sizes of the droplets observed through TEM were consistent with those measured using dynamic light scattering. Additionally, the size distribution of the droplets was found to be heterogeneous, indicating the presence of certain aggregates. Overall, these results confirm that the microstructures of the emulsions varied depending on the composition of the layers surrounding the droplets.

#### 3.2.3 Atomic force microscopy

Atomic Force Microscopy (AFM) is a powerful imaging technique that can be used for the characterization and analysis of nanoemulsion systems. AFM provides high-resolution topographical information and mechanical properties of the nanoemulsion droplets at the nanoscale. The 2D atomic force microscopy (AFM) images for NeClO (Fig. 1G) and NeCiO (Fig. 1H) formulations were displayed a spherical shape of nanoparticles with smooth surface, while the 3D images (AFM) for NeClO (Fig. 1I) and NeCiO (Fig. 1J) showed the height and depth in a three-dimensional representation of the surface. The NeClO and NeCiO formulations showed an average particle size ranging from 100 to 300 nm in all cases. AFM also showed the graphical representations of mean diameter of NeClO (Fig. 1K) and NeCiO (Fig. 1L).

### 3.3 Characterization of dehydration of nanoemulsions

#### 3.3.1 The encapsulation efficiency (EE)

The content of surface oil and total oil in the powders was used to determine the extent of oil entrapment in the capsules. In the study, the powders were characterized by microencapsulation efficiency, which ranged from 86.28 % to 91.75 % for microencapsulated clove oil and cinnamon oil, respectively. The microencapsulation efficiency of essential oils is an important quality parameter that indicates the amount of oil successfully encapsulated through spray drying [29]. The efficiency is defined as the ratio of total oil in the final powder to the amount of surface oil in the powder. A similar results was reported by **de Barros Fernandes et al.** [28] in their investigation of rosemary essential oil microencapsulation through spray drying. They examined the effects of gum arabic, modified starch, and inulin on various properties of the powder."

#### 3.3.2 Microcapsules powder morphology

**Fig. 2:**
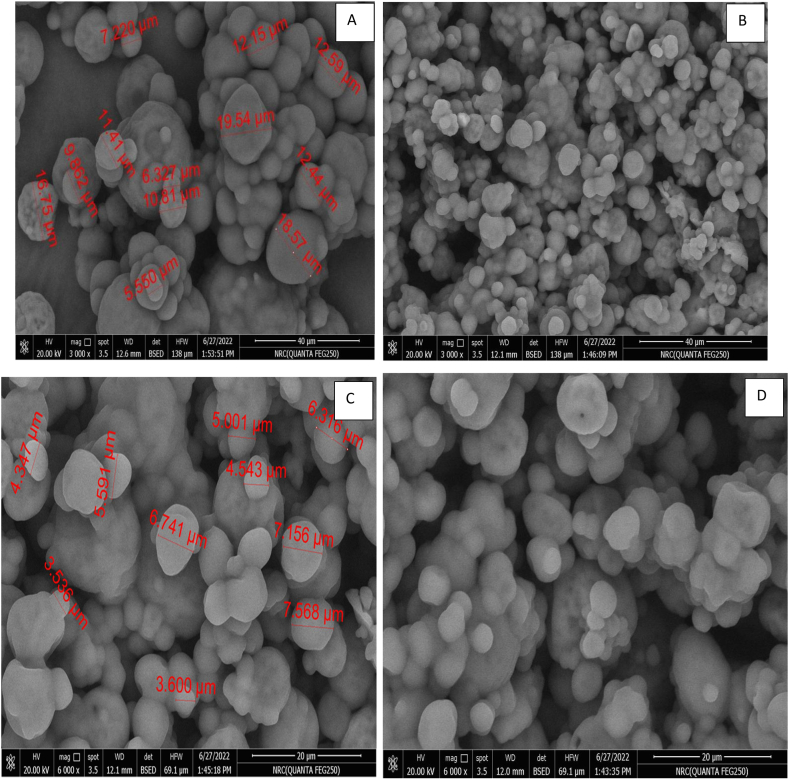
The morphology of microencapsulated clove oil (A & B) and cinnamon oil (C& D) using Scanning electrone microscope.

Fig. 2 illustrate the powder particles' surfaces for microencapsulated clove oil and cinnamon oil. It was apparent that MD, the primary encapsulating agent, was necessary to create several homogeneous capsules with smooth surfaces and an excellent spherical shape. In addition to other larger agglomerates of irregular shapes, the microparticles produced little wrinkles, smoother surfaces, and well-defined spherical microcapsules due to using gum Arabic in the sound material [68]**.** Considering the apparent correlation between particle size and viscosity, the higher viscosity of the feed emulsion may be the cause of the bigger particles or agglomerates. The occurrence of some indentations on the surface of spray-dried particles is typically attributed to particle shrinkage because of the rapid moisture loss and subsequent cooling. The continuous external particle surfaces had no breaks, cracks, or porosity. According to **Abdel-Razek et al.** [69] these characteristics are critical for ensuring active substances' considerable protection and retention.

### 3.4 Proximate composition of the innovative and commercial cakes

To avoid using wheat flour, which contains gluten and can cause sensitivity in certain people, and to take advantage of its nutritional benefits and antioxidant capabilities, this study employed regionally affordable and readily accessible rice flour and potato flour. Rice and potato flour are known to be energy-dense and have been suggested as potential substitutes for wheat flour in cake production due to their high energy content. According to a study by **Klunklin & Savage** [70], rice and potato flour can provide up to 10–20 times more energy than wheat flour, making them a valuable ingredient in gluten-free cake production. In addition to their energy content, rice and potato flour are rich sources of dietary protein, carbohydrates, and fibre. The results indicated in (Table 3) show that the innovative cake sample made with rice and potato flour had a higher content of carbohydrates, sugar, fibre, and protein (68.51, 12.87, 11.35, and 10.63 g/100 g sample, respectively) compared to the commercial cake sample made from wheat flour. The innovative cake sample had a lower total fat content (20.25 g/100 g) than the commercial sample. It is essential because high-fat diets have been linked to various health issues, including obesity and heart disease. Additionally, the commercial sample contained cholesterol, which can contribute to the development of atherosclerosis and other cardiovascular diseases.

**Table 3:** Nutrition Facts of cakes samples.

Nutrition Facts	Amount g/100 g sample	
	Commercial cake	Innovative cake
Calories (KJ/100g)	433.35 ± 3.55^b^	534.81 ± 3.38^a^
Total fat	22.11 ± 0.31^b^	20.25 ± 0.32^a^
Saturated fatty acid (g)	3.20 ± 0.19^b^	2.35 ± 0.17^a^
Monounsaturated fatty acid (g)	1.34 ± 0.05^b^	1.44 ± 0.06^a^
Polyunsaturated fatty acid (g)	17.12 ± 0.26^b^	16.09 ± 0.24^a^
Cholesterol (mg)	4.351 ± 0.14^b^	0.000 ± 0.00^a^
Total Carbohydrates (g)	51.42 ± 1.25^b^	68.51 ± 1.32^a^
Sugar (g)	2.10 ± 0.06^b^	12.87 ± 1.07^a^
Dietary Fiber (g)	5.65 ± 0.15^b^	11.35 ± 1.10^a^
Total Protein (g)	7.17 ± 0.27^b^	10.63 ± 0.58^a^

In each row, typical letters indicate a non-significant difference while atypical letters mean significant difference at 0.05 probability. The data are stated as average ± standard error.

The driving force for the increased consumption of bakery products from wheat flour has led to innovation in preparing meals with oats, bran, seeds, etc., that are more nutritious to attract health-conscious consumers [71]**.**

### 3.5 Vitamins content in cake samples

The contents of thiamine and riboflavin in the novelty and commercial cake samples in (Table 4) were similar to previous reports [72]. They were higher in the innovative cake sample at 0.82 and 0.54 mg/100g, respectively. As for the values of vitamin A and C, they were also higher in the innovative cake sample than the commercial cake: 1038.24 μg/100 gm and 0.27 mg/100 gm of sample, respectively, and it was higher in the content of riboflavin - 0.54 mg/100g of sample. The content of Niacin showed a significant difference between the samples, with higher values (P < 0.05) compared to the commercial cake.

**Table 4:** The vitamins’ estimate per 100g of cakes samples.

Vitamins	Amount (mg) per 100g sample	
	Commercial cake	Innovative cake
Vitamin A (mcg)	853.11 ± 9.81^b^	1038.24 ± 11.35^a^
Thiamin Vitamin B-1 (mg)	0.13 ± 0.02^b^	0.82 ± 0.04^a^
Riboflavin Vitamin B-2 (mg)	0.14 ± 0.01^b^	0.54 ± 0.02^a^
Vitamin C (Ascorbic Acid) (mg)	0.11 ± 0.02^b^	0.27 ± 0.02^a^
Niacin Vitamin B-3(mg)	0.91 ± 0.12^b^	2.08 ± 0.18^a^
Vitamin E (mg)	0.73 ± 0.18^b^	1.03 ± 0.15^a^

In each row, typical letters indicate a non-significant difference while atypical letters mean significant difference at 0.05 probability. The data are stated as average ± standard error.

### 3.6 Minerals analysis of cakes samples

Minerals (iron, calcium, zinc, sodium, potassium, and magnesium) were investigated for innovative cakes (gluten-free and fortified with innovative probiotic cream) compared to commercial cake samples because these minerals are important for health and potato and rice flour can be a source. The calcium content in the novelty cake of 63.11 mg/100g (Table 5) was higher than that described in the commercial cake sample of 47.25 mg/100g. Calcium is one of the more difficult minerals to reach the recommended daily intake, confirming the high nutritional value of innovative cake samples, which meet the daily needs of consumers.

**Table 5:** Minerals analysis for 100g of cakes samples.

Minerals	Amount (mg) per 100g sample	
	Commercial cake	Innovative cake
Calcium (Ca)	47.25 ± 3.54^b^	63.11 ± 3.68^a^
Magnesium (Mg)	21.54 ± 2.17^b^	36.82 ± 2.46^a^
Potassium (K)	68.22 ± 3.65^b^	79.55 ± 3.62^a^
Iron (Fe)	1.52 ± 0.14^b^	2.69 ± 0.21^a^
Zinc (Zn)	0.14 ± 0.01^b^	0.85 ± 0.02^a^
Sodium (Na)	298.35 ± 6.81^b^	364.43 ± 9.27^a^

In each row, typical letters indicate a non-significant difference while atypical letters mean significant difference at 0.05 probability. The data are stated as average ± standard error.

The iron contents of the innovative cake sample were within the range described in the literature [73]**.** Iron contents differed in all samples and were higher in the innovative cake sample than the commercial cake, 2.69 and 1.52 mg/100 g, respectively, which confirmed the fortification of the innovative product with iron.

The zinc content was also within the range described in the literature, and the zinc values obtained for the novelty cake sample were 0.85 mg/100g and eight times higher than those found for the commercial cake sample. It is known that zinc is essential in many metabolic pathways, and its deficiency leads to increased susceptibility to infections, delayed growth, and reproductive problems.

### 3.7 Total phenolic content (TPC) and total flavonoid content (TFC)

The total phenolic content (TPC) and total flavonoids (TFC) of the innovative cake sample were 1827.23 mg Gallic acid equivalents (GAE) (mg/100g dry extract) and 94.13 mg quercetin equivalents (QE) (mg/100g), respectively (Table 6). The TPC value of the commercial cake sample was less than 598.17 mg GAE/100 g, while the TFC was 41.33 mg QE/100 g. It is noted from the results the effect of the ingredients used in the preparation of innovative cakes on TPC and TFC. The high content of polyphenols in products reduces the degradation of these biologically active compounds during storage and presentation to the consumer, which prolongs the shelf life of those products rich in phenolics and their derivatives [74]**.**

**Table 6:** Total phenolic content (TPC) and total flavonoid content (TFC) of cake samples.

Cake types	TPC (mg) GAE/100 g sample	TFC (mg) QE/100 g sample
Commercial cake	598.17 ± 10.51^b^	41.33 ± 3.88^b^
Innovative cake	1827.23 ± 12.65^a^	94.13 ± 4.57^a^

In each column, typical letters indicate a non-significant difference while atypical letters mean significant difference at 0.05 probability. The data are stated as average ± standard error.

### 3.8 Antioxidant activity of cake samples

The antioxidant activity of cake samples for polyphenols is due to their ability to donate hydrogen atoms or free electrons. The free radical assay DPPH˙ was evaluated for antioxidant activity, which was found to be high in the innovative cake sample; it was 76.03 % antioxidants, while it was 13.54 % for the commercial cake sample. Increased radical scavenging activity of DPPH can be defined as antioxidant activity [75].

Reducing power indicates electron donation and is an essential mechanism for determining the antioxidant activity of cake samples, especially for phenolic compounds. The presence of reductions indicates that Fe3+ will reduce, and the reduction of antioxidant capacity indicates its antioxidant activity.

The FRAP test is usually used to measure the antioxidant capacity of innovative and commercial cake samples, as shown in Table 7.

**Table 7:** Antioxidant activity of cake samples.

Samples	DPPH %	FRAP %
Commercial cake	13.54 ± 2.59^a^	63.41 ± 4.85^b^
Innovative cake	76.03 ± 3.81^b^	132.63 ± 6.47^a^

In each column, typical letters indicate a non-significant difference while atypical letters mean significant difference at 0.05 probability. The data are stated as average ± standard error.

The highest antioxidant activity related to the innovative cake sample was 132.63 μmol Fe2+/g, while the lowest activity was in the commercial cake sample at 63.41 μmol Fe2+/g, which is due to the oils of cloves and cinnamon encapsulated in nanometer capsules and present in the ingredients of the innovative gluten-free cake dough.

### 3.9 Sensory evaluation of cake samples

Sensory evaluation is a critical stage for innovative cake samples and product functional property improvement studies because products aimed at consumers must first have sensory appeal to them [76]. Sensory traits, including colour, taste, mouth feel, flavour and general acceptance of innovative cake samples, were evaluated compared to the commercial sample. A summary of the mean sensory trait scores is presented in Table 8. For each attribute, scores were made on a scale of 1–10 on the hedonic scale. The innovative cake sample was better in colour, taste, flavour, and overall acceptability than the other formulations compared to the control (commercial) sample. However, consumer preference in terms of oral feeling was close in both the innovator and control samples.

**Table 8:** Sensory evaluation of cake samples.

Samples	Color	Taste	Mouth feel	Flavor	Overall acceptability
**The commercial cake**	7.5 ± 0.8^a^	7.2 ± 0.9^a^	8.0 ± 0.9^a^	8.1 ± 0.8^a^	7.70 ± 0.8^a^
**The innovative cake**	9.5 ± 1.0^b^	9.0 ± 0.8^b^	8.5 ± 1.0^a^	8.7 ± 0.9^a^	8.93 ± 1.1^a^

In each column, typical letters indicate a non-significant difference while atypical letters mean significant difference at 0.05 probability. The data are stated as average ± standard error.

### 3.10 Viability of probiotics during cake storage

**Fig. 3:**
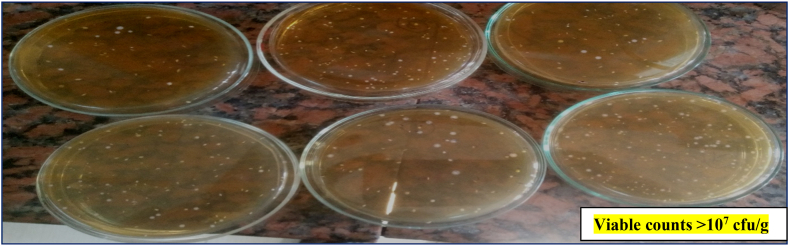
Viable probiotic counts in MRS agar after 10 days of cake storage.

The endowing of cake with probiotic properties provides a variety in food selection and potentially improves human health. The data presented in Table 9 and Fig. 3 showed the viable counts (log CFU/g) of probiotics in cake samples during storage. At zero time, the viable counts of probiotic strains were recorded at high levels (13.15 log CFU/g), and then the counts were gradually decreased by one log cycle after five days and by two log cycles (11.21 log CFU/g) at the end of the storage period. The counts of probiotics in the resultant cake were still above the threshold for therapeutic effects of 107 cfu/g [77]. Our results were in harmony with those obtained by **Arslan-Tontul et al.** [78]. They mentioned that acceptable probiotic counts in bakery products could be achieved when probiotics are added to the cake after baking or using double-layered microcapsules that increase the survivability of probiotic microorganisms during the cake-baking process and storage.

**Table 9:** The viable counts (log CFU/g) of probiotic lactobacilli in cake samples during storage.

	Storage time (days)			
	Fresh	5	7	10
Counts of probiotics (log CFU/g)	13.15 ± 0.03^a^	12.7 ± 0.12^b^	11.65 ± 0.21^c^	11.21 ± 0.22^cd^

In each row, typical letters indicate a non-significant difference while atypical letters mean significant difference at 0.05 probability. The data are stated as average ± standard error.

### 3.11 The protective effect of the innovative cake against liver injury induced by thioacetamide

Thioacetamide dose induction did not cause any mortality in animals. Also, feeding on the prepared cake did not cause animal mortality. Feeding on the prepared cake did not lead to clear toxicity, inferred by liver and kidney functions. On the contrary, feeding on cake reduced the rise in liver and kidney functions caused by treatment with TAA. The nutritional parameters (Table 10) showed a significant decrease (P ≤ 0.05) in final body weight for the TAA group. However, no significant changes existed between the negative group and rats fed on the prepared cake. Feeding on the prepared cake caused a corrective attitude in final body weight and body weight gain compared to rats of the positive control group.

**Table 10:** Effect of the prepared cake on the nutritional parameters.

	Initial body weight (g)	Final body weight (g)	Body weight gain (g)	Food intake (g)
**Control negative**	124.25 ± 1.5^a^	391.5 ± 1.2^a^	267.75 ± 1.5^a^	1462.5 ± 6.4^a^
**Control positive (Thioacetamide)**	124.5 ± 2.6^a^	350.75 ± 4.3^c^	226.25 ± 2.9^c^	1430.6 ± 5.2^b^
**The prepared cake + Thioacetamide**	125.75 ± 3.7^a^	363.5 ± 12.7^a^	237.75 ± 10.0^b^	1462.0 ± 4.8^a^

In each row, typical letters indicate a non-significant difference while atypical letters mean significant difference at 0.05 probability. The data are stated as average ± standard error.

Table 11 indicates that TAA induced hepatotoxicity with significant evaluation in serum level of specific liver enzymes AST, ALT, and ALP of the positive group compared to the other groups. Increased liver enzymes, AST, ALT, and ALP, are good indicators of liver dysfunction and necrosis. In addition, there were significant elevations in total protein, albumin and A/G ratio for the positive control group compared to the negative control group. Feeding on the prepared cake for eight weeks produced a significant (p ≤ 0.05) reduction in the levels of these enzymes. Also, feeding on the prepared cake suppressed the total protein, albumin, and A/G ratio elevations.

**Table 11:** Effect of the prepared cake on the nutritional parameters.

	Control negative	Control positive Thioacetamide	The prepared cake + Thioacetamide
AST (U/L)	37.28 ± 0.15^c^	123.75 ± 2.47^a^	78.50 ± 1.15^b^
ALT (U/L)	29.28 ± 0.07^c^	72.90 ± 0.76^a^	55.60 ± 1.56^b^
ALP (U/L)	70.29 ± 0.17^c^	105.02 ± 0.56^a^	78.60 ± 1.72^b^
Total protein (g/dl)	5.30 ± 0.19^b^	9.23 ± 0.02^a^	4.58 ± 0.10^c^
Albumin (g/dl)	3.33 ± 0.39^b^	7.08 ± 0.04^a^	2.60 ± 0.14^c^
A/G Ratio	1.66 ± 0.14^b^	3.29 ± 0.06^a^	1.33 ± 0.21^c^
Creatinine (mg/dl)	0.78 ± 0.08^c^	2.55 ± 0.05^a^	0.94 ± 0.05^b^
Urea (mg/dl)	34.32 ± 0.08^c^	48.70 ± 0.14^a^	40.45 ± 3.08^b^
Glucose (mg/dl)	91.65 ± 0.28^ab^	90.39 ± 5.34^b^	10.55 ± 0.24^b^
Hb (mg/dl)	15.38 ± 0.35^a^	97.64 ± 4.77^a^	12.12 ± 1.66^b^

In each column, typical letters indicate a non-significant difference while atypical letters mean significant difference at 0.05 probability. The data are stated as average ± standard error.

It was shown in Table (11) that renal impairment was observed in the TAA group, which was indicated by the significantly elevated urea and creatinine. The urea and creatinine values were significantly less for the rats fed on the prepared cake than for those of the positive group.

Table 12 indicates that TAA causes oxidative stress via the generation of reactive oxygen species (ROS) with significant elevation in liver MDA level and decrease in liver catalase, GPx, SOD and total antioxidant capacity of the positive control group compared to the other groups. ROS are an important inducer of liver fibrosis. Resultant lipid peroxidation causes tissue necrosis and inflammation, promoting the progression of tissue fibrogenesis. Feeding on the prepared cake suppressed the elevation in liver MDA level and decreased liver catalase, GPx, SOD and total antioxidant capacity.

**Table 12:** Effect of the prepared cake on the oxidative markers.

	Control negative	Control positive Thioacetamide	The prepared cake + Thioacetamide
MDA (nmol/g)	1.16 ± 0.12^b^	1.62 ± 0.19^a^	1.35 ± 0.03^b^
Total Antioxidant Capacity %	75.82 ± 1.60^b^	22.13 ± 0.24^c^	78.54 ± 2.04^a^
Liver catalase (U/g)	575.18 ± 4.14^a^	353.59 ± 2.48^c^	427.27 ± 1.47^b^
Liver GPX (U/g)	1116.86 ± 2.58^b^	616.40 ± 42.69^c^	2366.49 ± 88.76^a^
Liver SOD (U/g)	979.90 ± 6.80^a^	156.590 ± 8.89^c^	464.005 ± 7.61^b^

In each column, typical letters indicate a non-significant difference while atypical letters mean significant difference at 0.05 probability. The data are stated as average ± standard error.

Table 13 indicates that rats treated with only TAA recorded CD8 and IL-6 values significantly higher than those of the normal control group. While CD4 and INF-ᵧ values significantly decreased in rats treated with only TAA compared to the normal control group. Rats fed on the diet supplemented with the prepared cake recorded CD8 and IL-6 values less than those treated with only TAA. CD4 and INF-ᵧ values in rats fed on the diet supplemented with the prepared cake were significantly higher than in the positive control group.

**Table 13:** Effect of the prepared cake on the immunological markers.

	Control Negative	Control positive Thioacetamide	The prepared cake + Thioacetamide
CD4 (pg/ml)	756.50 ± 3.80^c^	651.67 ± 3.67^a^	731.67 ± 4.29^b^
CD8 (pg/ml)	372.00 ± 4.10^a^	385.42 ± 2.18^b^	374.17 ± 1.96^a^
CD4/CD8 ratio	2.04 ± 0.03^c^	1.69 ± 0.01^a^	1.96 ± 0.02^b^
INF-gamma (pg/ml)	28.73 ± 0.31^c^	22.42 ± 0.66^a^	25.63 ± 0.72^b^
IL-6 (pg/ml)	25.33 ± 1.45^a^	93.13 ± 1.97^c^	38.10 ± 0.84^b^

In each column, typical letters indicate a non-significant difference while atypical letters mean significant difference at 0.05 probability. The data are stated as average ± standard error.

Table 14 indicates that the rats treated with only TAA recorded significantly higher cholesterol, LDL, and triglyceride values than those of the normal control group. While HDL value significantly decreased in the rats treated with only TAA compared to the normal control group. The results of lipid profile indices showed that TAA caused dyslipidemia. The rats fed on the diet supplemented with the prepared cake recorded less cholesterol, LDL, and triglyceride values than those treated with only TAA. HDL value in the rats fed on the diet supplemented with the prepared cake was significantly higher than in the positive control group.

**Table 14:** Effect of the prepared cake on the lipid profile.

	Control Negative	Control positive Thioacetamide	The prepared cake + Thioacetamide
Cholesterol (mg/dl)	116.170 ± 1.702^b^	125.6850 ± 1.093^a^	119.065 ± 2.83^b^
LDL (mg/dl)	34.953 ± 1.572^c^	80.715 ± 1.508^a^	46.515 ± 4.042^b^
HDL (mg/dl)	58.757 ± 0.542^a^	25.457 ± 0.237^c^	52.155 ± 2.73^b^
Triglycerides (mg/dl)	112.300 ± 0.15^a^	97.552 ± 1.540^b^	101.984 ± 5.601^b^
VLDL (mg/dl)	22.460 ± 0.031^a^	19.510 ± 0.307^b^	20.396 ± 1.120^b^
TC/HDL	1.977 ± 0.027^c^	4.937 ± 0.075^a^	2.287 ± 0.1268^b^
TG/HDL	1.911 ± 0.016^b^	3.831 ± 0.045^a^	1.961 ± 0.175^b^
LDL/HDL	0.594 ± 0.0269^c^	3.171 ± 0.080^a^	0.895 ± 0.108^b^
Atherogenic coefficient	0.977 ± 0.027^c^	3.9374 ± 0.075^a^	1.287 ± 0.126^b^
AIP	0.281 ± 0.003^b^	0.583 ± 0.005^a^	0.2912 ± 0.0396^b^

In each column, typical letters indicate a non-significant difference while atypical letters mean significant difference at 0.05 probability. The data are stated as average ± standard error.

## 4 Discussion

According to the GC-MS results of clove and volatile cinnamon oils, the major components were eugenol and cinnamaldehyde in clove and volatile cinnamon oils. **Haro-González et al.** [57] also found that eugenol is the primary compound, accounting for at least 50 %. The remaining 10–40 % comprises eugenyl acetate, β-caryophyllene, and α-humulene. **Xavier et al.** [60] reported that cinnamon essential oil has biological activities, including anticancer, antidiabetic, antioxidant, anti-inflammatory and anti-human immunodeficiency virus. Such effects may be attributed to the unique secondary metabolic, including cinnamaldehyde, eugenol, cinnamyl acetate, methyl cinnamate, (E)-caryophyllene, and linalool.

The use of rice flour and sweet potatoes as a source of starch to produce a gluten-free cake that causes allergies in some people and to take advantage of the nutritional benefits and antioxidant capacity, and its economic cost is lower, and it is available throughout the year. There were significant differences in statistical indications for the innovative cake samples compared to the control (commercial) sample.

The innovative cake sample made with rice and potato flour had more carbohydrates, sugar, fibre and protein than the commercial cake sample made with wheat flour. The innovative cake sample also had a lower total fat content than the commercial sample. It is important because high-fat diets have been linked to many health problems, including obesity and heart disease. In addition, the commercial sample contained cholesterol, which can contribute to atherosclerosis and other cardiovascular diseases.

The total TPC and TFC of the innovative cake sample were higher than the commercial cake sample. The high polyphenols content in the products reduces the degradation of these bioactive compounds during storage and presentation to the consumer, which prolongs the shelf life of those products rich in phenols and their derivatives.

The antioxidant activity of cake samples of polyphenols is due to their ability to donate hydrogen atoms or free electrons. The antioxidant activity was found to be high in the innovative cake sample, thus its ability to increase the radical scavenging of DPPH. It is due to the nanometer-capsulated oils of clove and cinnamon found in our innovative gluten-free cake batter ingredients.

Sensory evaluation is a critical stage for innovative cake samples and studies to improve the product's functional properties. Sensory features, including colour, taste, mouth feel, flavour and general acceptance of innovative cake samples, were evaluated compared to the commercial sample. The innovative cake sample was better in colour, taste, flavour and general acceptance for the other formulations than the control (commercial) sample.

Probiotics, clove and cinnamon essential oils, as liver-protective and immune-enhancing agents, were utilized in the current study to prepare a functional cake, one of the popular products that are easy to manufacture and handle. To avoid using wheat flour, which contains gluten and can cause sensitivity in certain people, and to take advantage of its nutritional benefits and antioxidant capabilities, this study employed regionally affordable and readily accessible rice and potato flour.

The findings of the present study indicated that TAA resulted in a reduction in body weight, which was also confirmed by **Al-Bader et al.** [79]**.** On the contrary, the weight gain in rats fed cake was greater than in rats treated with TAA which indicates the ability of cake to reduce the harmful effects of TAA. **Shareef et al.** [80] **and Lim et al.** [81] reported that TAA produced liver fibrosis and had a hepato-carcinogenesis effect. The hepato-protective effect of the prepared cake may be attributed to the orange sweet potatoes that restored the changes elicited by TAA assault on liver markers, and hepatic necrosis may be due to the antioxidant compounds, mainly beta-carotene [82]**.** In addition, cinnamaldehyde, the main compound in cinnamon oil, positively affected the hepatocellular carcinoma induced by TAA, as reported by **Abd El Salam et al.** [83]**.** Probiotics also have therapeutic effects on liver illnesses because they lessen oxidative stress, inflammation, and fibrosis, all of which contribute to the developing of several different liver diseases. Probiotics largely affect how the normal gut flora behaves and is composed. Intestinal homeostasis is restored by maintaining a healthy microbiota, which enhances ATP production and minimizes adverse metabolic effects on the liver (**Dewanjee et al.** [20]**.** The antioxidant effect of the prepared cake may be attributed to the presence of sweet potato, which is considered a valuable source of antioxidants, including vitamin A (a potent antioxidant that reduces free radical damage and aids in the battle against inflammation), anthocyanins that may be able to reduce any potential health problems brought on by oxygen radicals and beta carotene. A previous study found that cupcakes prepared from lyophilized sweet potato powder had an antioxidant effect and improved oxidative markers in rats [84]**.** By interfering with ROS-producing enzymes such as NADPH oxidases, CYPs, and cyclooxygenases, probiotics also reduce the production of ROS [82]. **Haro-González et al.** [57] reported that clove oil possesses antioxidant power due to its constituent, mainly eugenol, acetate and β-caryophyllene. **Wang et al.** [85] reported that the antioxidants eugenol and cinnamaldehyde are powerful in preventing diseases caused by free radicals, including cancer, inflammatory problems, type 2 diabetes, cardiovascular disease, neurological disorders, and periodontal disease. Like human alcoholic fibrogenesis, TAA causes liver fibrogenesis and inflammation, increasing the pro-inflammproinflammatory [86]. In this study, the prepared cake intake significantly ameliorated TAA-induced impairment in the immune status, as manifested by diminishing the rise of CD8 and increasing CD4. The antioxidant and anti-inflammatory effects of the prepared cake may be involved in the amelioration effect of TAA-induced hepatic inflammation. Probiotics have immune-modulatory and microbiota-balancing properties. They defend against pathogens, prevent an excessive effector T cell response, lower nuclear factor-kappa B (NF-kB) signalling and abnormal basal keratinocyte apoptosis, and change the mucosal response to produce anti-inflammatory cytokines [87]. Polyphenols, flavonoids, and carotenoids are among the functional components in sweet potatoes that display a range of bioactivities, including antioxidants, immune system modulation, and hepatoprotective properties [88]. Oxid stress and inflammation activate hepatic stellate cells (HSCs), and this process leads to fibrogenesis. It is well known that activated HSCs express cytokines that are important mediators of fibrogenesis in the context of the inflammatory cascade. TGF-1β causes the release of the proinflammaproinflammatory NF-α from HSCs, which in turn triggers the release of other proinflammaproinflammatory like IL-1β to start inflammatory reactions [89]. In this study, by reducing the rise of IL-6, the prepared cake treatment greatly reduced TAA-induced hepatic inflammation. Importantly, multiple experiments revealed that sweet potatoes have potent anti-inflammatory properties. **Jiang et al.** [90] **and Escobar-Puentes et al.** [91] reported that the phytochemicals in sweet potato, including polysaccharides, anthocyanins and polyphenols, had anti-inflammatory activities and diminished TNF-α, IL-1β, and IL-6 and reduced the activation of NF-κβ in RAW 264.7 cells induced by LPS. In addition, essential oils, especially clove oil, have antiviral, anti-inflammatory, and immune-modulatory properties. Clove oil has anti-inflammatory properties and has been shown to protect the lungs against lipopolysaccharide-induced acute injury [92]**.** Also, **Yadalam et al.** [93] **and Asif et al.** [92] recommended using clove and cinnamon essential oil (Eugenol 20μ/ml) and (Cinnamaldehyde 1 μg/ml) in treatment. Alongside with **Roth-Walter et al.** [94], the ability of cinnamaldehyde to prevent nuclear factor-kB activation in immune cells is referred to as one of its anti-inflammatory qualities. In primary and immortalized immune cells, cinnamaldehyde treatment inhibited cell viability and proliferation and caused apoptosis in a dose-dependent manner. Dyslipidemia is associated with metabolic syndrome also, and it is considered a common complication which had a critical role in increases in mortality and severity for patients [95]. The hypolipidemic effect of the prepared cake may be due to fibre, antioxidants, phytochemicals and vitamin content [96,97]. Recent research has indicated that lipid abnormalities may be prevented and treated with therapies that target gut bacteria. Probiotic strain supplements are typically prepared with species that are Generally Recognized as safe. Such species are also commonly found in the gastrointestinal tracts of healthy individuals. Scientific interest in the relationship between intestinal microbiota and cardiovascular health showed a hypocholesterolemic effect. According to **Wang et al.** [98]**,** probiotic consumption could help alleviate blood lipids' dysregulation and decrease cholesterolemia.

## 5 Conclusion

The study proved that the innovative cake fortified with probiotics, prepared from sweet potato and rice flour and flavoured with micro-encapsulated clove oils and cinnamon oils, has nutritional value, sensory ability, and antioxidant properties, and is gluten-free compared to commercial cake, which improves the quality and nutritional characteristics of that new product, which affects the longevity of Consumer acceptance, in addition to increasing the shelf life of these products and offering them in the market. However, the study suggests that the prepared cake has potential liver-protective, immune-enhancing, and hypolipidemic effects, which may be attributed to the combination of probiotics, clove and cinnamon essential oils, and sweet potatoes. These findings may have implications for developing functional foods that can improve health outcomes, especially in individuals with liver diseases or dyslipidemia. As well as further research is needed to confirm these findings and to determine the underlying mechanisms.

## Data availability statement

The data that support the findings of this study are available within the article.

## CRediT authorship contribution statement

**Manal M. Ramadan:** Conceptualization, Investigation, Project administration, Supervision. **Eman F. El Haggar:** Formal analysis, Methodology, Writing - original draft. **Rasha S. Mohamed:** Formal analysis, Methodology, Visualization, Writing - original draft. **Khaled F. Mahmoud:** Conceptualization, Methodology, Validation, Visualization. **Ahmed M. Mabrouk:** Methodology, Writing - original draft. **Amal G. Hussien:** Methodology. **Abeer E. Mahmoud:** Writing - original draft. **Ola A.M. Mohawed:** Formal analysis, Methodology, Validation. **Tamer M. El-Messery:** Visualization, Writing - review & editing.

## Declaration of competing interest

The authors declare that they have no known competing financial interests or personal relationships that could have appeared to influence the work reported in this paper.

## References

[1] Hill C., Guarner F., Reid G., Gibson G.R., Merenstein D.J., Pot B., Sanders M.E. (2014). The International Scientific Association for Probiotics and Prebiotics consensus statement on the scope and appropriate use of the term probiotic. Nat. Rev. Gastroenterol. Hepatol..

[2] Gibson G.R., Hutkins R.W., Sanders M.E., Prescott S.L., Reimer R.A., Salminen S.J., Reid G. (2017).

[3] Gill A.O., Holley R.A. (2004). Mechanisms of bactericidal action of cinnamaldehyde against Listeria monocytogenes and of eugenol against L. monocytogenes and Lactobacillus sakei. Appl. Environ. Microbiol..

[4] Nazzaro F., Fratianni F., Coppola R., De Feo V. (2017). Essential oils and antifungal activity. Pharmaceuticals.

[5] McClements D.J. (2018). Recent developments in encapsulation and release of functional food ingredients: delivery by design. Curr. Opin. Food Sci..

[6] El Barnossi A., Moussaid F., Housseini A.I. (2021). Tangerine, banana and pomegranate peels valorisation for sustainable environment: a review. Biotechnology Reports.

[7] Savic I.M., Savic Gajic I.M. (2022). Determination of physico-chemical and functional properties of plum seed cakes for estimation of their further industrial applications. Sustainability.

[8] Horstmann S.W., Lynch K.M., Arendt E.K. (2017). Starch characteristics linked to gluten-free products. Foods.

[9] Samadder A., Abraham S.K., Khuda-Bukhsh A.R. (2016). Nanopharmaceutical approach using pelargonidin towards enhancement of efficacy for prevention of alloxan-induced DNA damage in L6 cells via activation of PARP and p53. Environ. Toxicol. Pharmacol..

[10] Abdulqahar F.W., El-Messery T.M., Zaky A.A., El-Said M.M. (2022). In vitro digestibility of Aucklandia costus-loaded nanophytosomes and their use in yoghurt as a food model. Food Biosci..

[11] Samadder A., Das S., Das J., Khuda-Bukhsh A.R. (2013). Relative efficacies of insulin and poly (lactic-co-glycolic) acid encapsulated nano-insulin in modulating certain significant biomarkers in arsenic intoxicated L6 cells. Colloids Surf. B Biointerfaces.

[12] Samadder A., Tarafdar D., Abraham S.K., Ghosh K., Khuda-Bukhsh A.R. (2017). Nano-pelargonidin protects hyperglycemic-induced L6 cells against mitochondrial dysfunction. Planta Med..

[13] Abdel-Wahhab M.A., El-Nekeety A.A., Mohammed H.E., El-Messery T.M., Roby M.H., Abdel-Aziem S.H., Hassan N.S. (2021). Synthesis of encapsulated fish oil using whey protein isolate to prevent the oxidative damage and cytotoxicity of titanium dioxide nanoparticles in rats. Heliyon.

[14] Das J., Samadder A., Mondal J., Abraham S.K., Khuda-Bukhsh A.R. (2016). Nano-encapsulated chlorophyllin significantly delays progression of lung cancer both in in vitro and in vivo models through activation of mitochondrial signaling cascades and drug-DNA interaction. Environ. Toxicol. Pharmacol..

[15] Abd El-Gawad A., Kenawy M.A., El-Messery T.M., Hassan M.E., El-Nekeety A.A., Abdel-Wahhab M.A. (2023). Fabrication and characterization of bee venom-loaded nanoliposomes: enhanced anticancer activity against different human cancer cell lines via the modulation of apoptosis-related genes. J. Drug Deliv. Sci. Technol..

[16] Messina A., Luce E., Hussein M., Dubart-Kupperschmitt A. (2020). Pluripotent-stem-cell-derived hepatic cells: hepatocytes and organoids for liver therapy and regeneration. Cells.

[17] Gao B. (2016). Basic liver immunology. Cell. Mol. Immunol..

[18] FAO/WHO (2002). http://www.who.int/foods.

[19] Jantararussamee C., Rodniem S., Taweechotipatr M., Showpittapornchai U., Pradidarcheep W. (2021). Hepatoprotective effect of probiotic lactic acid bacteria on thioacetamide-induced liver fibrosis in rats. Probiotics and Antimicrobial Proteins.

[20] Dewanjee S., Dua T.K., Paul P., Dey A., Vallamkondu J., Samanta S., De Feo V. (2022). Probiotics: evolving as a potential therapeutic option against acetaminophen-induced hepatotoxicity. Biomedicines.

[21] Raj T., Chandrasekhar K., Kumar A.N., Kim S.H. (2022). Recent biotechnological trends in lactic acid bacterial fermentation for food processing industries. Systems Microbiology and Biomanufacturing.

[22] Gulec Peker E.G., Kaltalioglu K. (2021). Cinnamaldehyde and eugenol protect against LPS‐stimulated oxidative stress and inflammation in Raw 264.7 cells. J. Food Biochem..

[23] Yakhchali M., Taghipour Z., Ardakani M.M., Vaghasloo M.A., Vazirian M., Sadrai S. (2021). Cinnamon and its possible impact on COVID-19: the viewpoint of traditional and conventional medicine. Biomed. Pharmacother..

[24] Paidi R.K., Jana M., Raha S., McKay M., Sheinin M., Mishra R.K., Pahan K. (2021). Eugenol, a component of holy basil (tulsi) and common spice clove, inhibits the interaction between SARS-CoV-2 spike S1 and ACE2 to induce therapeutic responses. J. Neuroimmune Pharmacol..

[25] Purkait S., Bhattacharya A., Bag A., Chattopadhyay R.R. (2021). TLC bioautography–guided isolation of essential oil components of cinnamon and clove and assessment of their antimicrobial and antioxidant potential in combination. Environ. Sci. Pollut. Control Ser..

[26] Adams R.P. (2017).

[27] Balci‐Torun F. (2023). Encapsulation of Origanum onites essential oil with different wall material using spray drying. Phytochem. Anal..

[28] de Barros Fernandes R.V., Marques G.R., Borges S.V., Botrel D.A. (2014). Effect of solids content and oil load on the microencapsulation process of rosemary essential oil. Ind. Crop. Prod..

[29] El-Messery T.M., Altuntas U., Altin G., Özçelik B. (2020). The effect of spray-drying and freeze-drying on encapsulation efficiency, in vitro bioaccessibility and oxidative stability of krill oil nanoemulsion system. Food Hydrocolloids.

[30] Bennion E.B., Bamford G.S.T. (1997).

[31] Horwitz W. (2000).

[32] Rangnna S. (2007). Handbook of analysis and quality control for fruits and vegetable products. Tata Mcgrawhill.

[33] Atwater W.O. (1902).

[34] Lee S.C., Prosky L., Vries J.W.D. (1992). Determination of total, soluble, and insoluble dietary fiber in foods—enzymatic-gravimetric method, MES-TRIS buffer: collaborative study. J. AOAC Int..

[35] Jahan S., Gosh T., Begum M., Saha B.K. (2011). Nutritional profile of some tropical fruits in Bangladesh: specially anti-oxidant vitamins and minerals. Bangladesh J. Med. Sci..

[36] Kirk S., Sawyer R. (1991).

[37] Mythili K., Reddy C.U., Chamundeeswari D., Manna P.K. (2014). Determination of total phenol, alkaloid, flavonoid and tannin in different extracts of Calanthe triplicata. J. Pharmacogn. Phytochem..

[38] Sharopov F.S., Wink M., Setzer W.N. (2015). Radical scavenging and antioxidant activities of essential oil components–An experimental and computational investigation. Nat. Prod. Commun..

[39] Lawless H.T., Heymann H. (2010).

[40] Lee S.Y., Vedamuthu E.R., Washam C.J., Reinbold G.W. (1974). An agar medium for the differential enumeration of yogurt starter bacteria. J. Food Protect..

[41] Hamdy S.M., Abdelmontaleb H.S., Mabrouk A.M., Abbas K.A. (2021). Physicochemical, viability, microstructure, and sensory properties of whole and skimmed buffalo set‐yogurts containing different levels of polydextrose during refrigerated storage. J. Food Process. Preserv..

[42] Reeves P.G., Nielsen F.H., Fahey G.C. (1993). AIN-93 purified diets for laboratory rodents: final report of the American Institute of Nutrition ad hoc writing committee on the reformulation of the AIN-76A rodent diet. The Journal of nutrition.

[43] Furtado K.S., Prado M.G., Aguiar e Silva M.A., Dias M.C., Rivelli D.P., Rodrigues M.A., Barbisan L.F. (2012). Coffee and caffeine protect against liver injury induced by thioacetamide in male Wistar rats. Basic Clin. Pharmacol. Toxicol..

[44] Drabkin D.L. (1949). The standardization of hemoglobin measurement. American J. Medical Sciences.

[45] Trinder P. (1969). Glucose assay: a colorimetric enzyme-kinetic method assay. Ann. Clin. Biochem..

[46] Rheinhold J.G., Seligron D. (1953).

[47] Reitman S. (1957). Liver enzymes (AST and ALT); Reitman and Frankel calorimetric method. Am J Uni Path.

[48] Doumas B.T., Watson W.A., Biggs H.G. (1997). Albumin standards and the measurement of serum albumin with bromocresol green. Clin. Chim. Acta.

[49] Larsin K. (1972). Creatinine assay by a reaction-kinetic approach. Clin Chem Acta.

[50] Fawcett J.K. (1960). Scott Je. A rapid and precise method for the determination of urea. J. Clin. Pathol..

[51] Ohkawa H., Ohishi W., Yagi K. (1979). Colorimetric method for determination of MDA activity. Biochemistry.

[52] Paglia D.E., Valentine W.N. (1967). Studies on the quantitative and qualitative characterization of erythrocyte glutathione peroxidase. J. Lab. Clin. Med..

[53] Beers R.F., Sizer I.W. (1952). A spectrophotometric method for measuring the breakdown of hydrogen peroxide by catalase. J. Biol. Chem..

[54] Nishikimi M., Rao N.A., Yogi K. (1972). Colorimetric determination of super oxide dismutase. Biochem. Bioph. Common.

[55] Watson D. (1960). A simple method for the determination of serum cholesterol. Clin. Chim. Acta.

[56] Burstein M.S.H.R., Scholnick H.R., Morfin R. (1970). Rapid method for the isolation of lipoproteins from human serum by precipitation with polyanions. Journal of lipid research.

[57] Schriewer H., Kohnert U., Assmann G. (1984).

[58] Megraw R.E., Dunn D.E., Biggs H.G. (1979). Manual and continuous-flow colorimetry of triacylglycerols by a fully enzymic method. Clinical chemistry.

[59] Haro-González J.N., Castillo-Herrera G.A., Martínez-Velázquez M., Espinosa-Andrews H. (2021). Clove essential oil (Syzygium aromaticum L. Myrtaceae): extraction, chemical composition, food applications, and essential bioactivity for human health. Molecules.

[60] Xavier J.K.A., Baia T.G.C., Alegria O.V.C., Figueiredo P.L.B., Carneiro A.R., Moreira E.C.D.O., da Silva J.K.R. (2022). Essential oil chemotypes and genetic variability of cinnamomum verum leaf samples commercialized and cultivated in the amazon. Molecules.

[61] Mutlu M., Bingol Z., Uc E.M., Köksal E., Goren A.C., Alwasel S.H., Gulcin İ. (2023). Comprehensive metabolite profiling of cinnamon (Cinnamomum zeylanicum) leaf oil using LC-HR/MS, GC/MS, and GC-FID: determination of antiglaucoma, antioxidant, anticholinergic, and antidiabetic profiles. Life.

[62] Villalobos-Castillejos F., Granillo-Guerrero V.G., Leyva-Daniel D.E., Alamilla-Beltrán L., Gutiérrez-López G.F., Monroy-Villagrana A., Jafari S.M. (2018). In Nanoemulsions.

[63] Premi M., Sharma H.K. (2017). Effect of different combinations of maltodextrin, gum Arabic and whey protein concentrate on the encapsulation behavior and oxidative stability of spray dried drumstick (Moringa oleifera) oil. Int. J. Biol. Macromol..

[64] Eratte D., Wang B., Dowling K., Barrow C.J., Adhikari B.P. (2014). Complex coacervation with whey protein isolate and gum Arabic for the microencapsulation of omega-3 rich tuna oil. Food Funct..

[65] Cansell M., Nacka F., Combe N. (2003). Marine lipid‐based liposomes increase in vivo FA bioavailability. Lipids.

[66] Mcclements D.J. (2005).

[67] Weinbreck F., Minor M., De Kruif C.G. (2004). Microencapsulation of oils using whey protein/gum Arabic coacervates. J. Microencapsul..

[68] Ramadan M.M., El‐Said M.M., El‐Messery T.M., Mohamed R.S. (2021). Development of flavored yoghurt fortified with microcapsules of triple omega 3‐6‐9 for preventing neurotoxicity induced by aluminum chloride in rats. J. Food Process. Preserv..

[69] Abdel-Razek A.G., Hassanein M.M., Ozçelik B., Baranenko D.A., El-Messery T.M. (2022). Omega fatty acid-balanced oil formula and enhancing its oxidative stability by encapsulation with whey protein concentrate. Food Biosci..

[70] Klunklin W., Savage G. (2018). Biscuits: a substitution of wheat flour with purple rice flour. Advances in Food Science and Engineering.

[71] Lambert J.L., LE‐Bail A., Zuniga R., VAN‐Haesendonck I., Vnzeveren E., Petit C., Ziobro R. (2009). The attitudes of European consumers toward innovation in bread; interest of the consumers toward selected quality attributes. J. Sensory Stud..

[72] Gomes L.D.O.F., Santiago R.D.A.C., Carvalho A.V., Carvalho R.N., Oliveira I.G.D., Bassinello P.Z. (2015). Application of extruded broken bean flour for formulation of gluten-free cake blends. Food Science and Technology.

[73] Gomes J.C., Oliveira Da Silva C., Costa N.M.B., Pirozi M.R. (2006). Development and characterization of common bean flour. Rev. Ceres.

[74] Ahmad-Qasem M.H., Barrajón-Catalán E., Micol V., Mulet A., García-Pérez J.V. (2013). Influence of freezing and dehydration of olive leaves (var. Serrana) on extract composition and antioxidant potential. Food Res. Int..

[75] Zhou K., Yu L. (2004). Effects of extraction solvent on wheat bran antioxidant activity estimation. LWT-Food science and Technology.

[76] Atef A.M.A.Z., Mostafa T.R., Al-Asklany S.A. (2011). Utilization of faba bean and cowpea flours in gluten free cake production. Australian Journal of Basic and Applied Sciences.

[77] Mabrouk A.M., Abd-Elgawad A.R., Abd El-Montaleb H.S. (2022). Effect of inulin and oat flour on viability of probiotics and physicochemical properties of reduced fat synbiotic ice cream. Br. Food J..

[78] Arslan-Tontul S., Erbas M., Gorgulu A. (2019). The Use of probiotic-loaded single-and double-layered microcapsules in cake production. Probiotics and antimicrobial proteins.

[79] Al‐Bader A., Mathew T.C., Khoursheed M., Asfar S., Al‐Sayer H., Dashti H.M. (2000). Thioacetamide toxicity and the spleen: histological and biochemical analysis. Anat. Histol. Embryol..

[80] Shareef S.H., Ibrahim I.A.A., Alzahrani A.R., Al-Medhtiy M.H., Abdulla M.A. (2022). Hepatoprotective effects of methanolic extract of green tea against Thioacetamide-Induced liver injury in Sprague Dawley rats. Saudi J. Biol. Sci..

[81] Lim J.Y., Jung W.W., Kim W., Moon K.S., Sul D. (2022). Nephrotoxicity evaluation and proteomic analysis in kidneys of rats exposed to thioacetamide. Sci. Rep..

[82] Wang L., Zhao Y., Zhou Q., Luo C.L., Deng A.P., Zhang Z.C., Zhang J.L. (2017). Characterization and hepatoprotective activity of anthocyanins from purple sweet potato (Ipomoea batatas L. cultivar Eshu No. 8). J. Food Drug Anal..

[83] Abd El Salam A.S.G., Samra Y.A., El-Shishtawy M.M. (2022). Cinnamaldehyde relieves induced hepatocellular carcinoma in rat model via targeting wnt/β-catenin pathway. Sci. Pharm..

[84] Hussein A.M., Mohamed R.S., Fouda K.A., Salama M.F., Hussein M.M. (2021). Functional cupcake for preventing vitamin A deficiency and correlated anemia and oxidative stress. Pakistan J. Biol. Sci.: PJBS.

[85] Wang Y., Wu Y., Wang Y., Xu H., Mei X., Yu D., Li W. (2017). Antioxidant properties of probiotic bacteria. Nutrients.

[86] Wallace M.C., Hamesch K., Lunova M., Kim Y., Weiskirchen R., Strnad P., Friedman S.L. (2015). Standard operating procedures in experimental liver research: thioacetamide model in mice and rats. Laboratory animals.

[87] Zanetta P., Ormelli M., Amoruso A., Pane M., Azzimonti B., Squarzanti D.F. (2022). Probiotics as potential biological immunomodulators in the management of oral lichen planus: what's new?. Int. J. Mol. Sci..

[88] Ayeleso T.B., Ramachela K., Mukwevho E. (2016). A review of therapeutic potentials of sweet potato: pharmacological activities and influence of the cultivar. Trop. J. Pharmaceut. Res..

[89] Alkreathy H.M., Esmat A. (2022). Lycorine ameliorates thioacetamide-induced hepatic fibrosis in rats: emphasis on antioxidant, anti-inflammatory, and STAT3 inhibition effects. Pharmaceuticals.

[90] Jiang T., Zhou J., Liu W., Tao W., He J., Jin W., Li Y. (2020). The anti-inflammatory potential of protein-bound anthocyanin compounds from purple sweet potato in LPS-induced RAW264. 7 macrophages. Food Res. Int..

[91] Escobar-Puentes A.A., Palomo I., Rodríguez L., Fuentes E., Villegas-Ochoa M.A., González-Aguilar G.A., Wall-Medrano A. (2022). Sweet potato (Ipomoea batatas L.) phenotypes: from agroindustry to health effects. Foods.

[92] Asif M., Saleem M., Saadullah M., Yaseen H.S., Al Zarzour R. (2020). COVID-19 and therapy with essential oils having antiviral, anti-inflammatory, and immunomodulatory properties. Inflammopharmacology.

[93] Yadalam P.K., Varatharajan K., Rajapandian K., Chopra P., Arumuganainar D., Nagarathnam T., Madhavan T. (2021). Antiviral essential oil components against SARS-CoV-2 in pre-procedural mouth rinses for dental settings during COVID-19: a computational study. Frontiers in chemistry.

[94] Roth-Walter F., Moskovskich A., Gomez-Casado C., Diaz-Perales A., Oida K., Singer J., Jensen-Jarolim E. (2014). Immune suppressive effect of cinnamaldehyde due to inhibition of proliferation and induction of apoptosis in immune cells: implications in cancer. PLoS One.

[95] Liu Y., Pan Y., Yin Y., Chen W., Li X. (2021). Association of dyslipidemia with the severity and mortality of coronavirus disease 2019 (COVID-19): a meta-analysis. Virol. J..

[96] Abd El-Rahman H.S.M., Abd-Elhak N.A., Zaki N.L. (2020). Hepatoprotective effects of mulberries and cape gooseberry on thioacetamide induced liver injury in rats. American Journal of Food and Nutrition.

[97] Chang G.R., Lin W.L., Lin T.C., Liao H.J., Lu Y.W. (2021). The ameliorative effects of saikosaponin in thioacetamide-induced liver injury and non-alcoholic fatty liver disease in mice. Int. J. Mol. Sci..

[98] Wang C., Zhang C., Li S., Yu L., Tian F., Zhao J., Zhai Q. (2020). Effects of probiotic supplementation on dyslipidemia in type 2 diabetes mellitus: a meta-analysis of randomized controlled trials. Foods.

